# On the Relation Between Procedural Learning and Syntactic Proficiency in Gifted Children

**DOI:** 10.1007/s10936-018-9611-6

**Published:** 2018-10-25

**Authors:** Sybren Spit, Judith Rispens

**Affiliations:** 1 Amsterdam Center for Language and Communication, University of Amsterdam, Amsterdam, The Netherlands; 2 University of Amsterdam, Spuistraat 134, 1012 VB Amsterdam, The Netherlands

**Keywords:** Giftedness, Object relative clauses, Procedural memory, Serial reaction time task

## Abstract

Gifted children are described as very talented children who achieve more than their age mates in one or more domains (Steiner and Carr in Educ Psychol Rev 15(3):215–246, 2003). These children potentially share a cognitive advantage enabling them to excel in language, but also in other domains. In the present study we explored whether gifted children have a relatively advanced procedural memory. We further investigated the relation between procedural memory and complex syntactic comprehension. 25 gifted children and as many non-gifted children between ages 8 and 13 were administered a serial reaction time (SRT) task and a relative clause comprehension task. Results from the SRT task showed no significant difference between gifted children and their TD peers, whereas gifted children showed significant better comprehension of object relative clauses. No significant correlations were found between the two tasks. There was thus no evidence that gifted children excel in procedural memory. Possibly some other factor, such as meta-linguistic knowledge or a beneficial social environment, contributed to their advanced linguistic comprehension.

## Introduction

When children are very talented and achieve or have the potential to achieve more than their age mates in one or more domains, they are often described as being gifted (Steiner and Carr 2003; Subotnik et al. 2011). These children excel in a wide range of areas, but may all share a cognitive advantage enabling them to do so. In this study we explore whether a relatively better developed procedural memory, which is involved in the implicit recognition of abstract rules in several domains, including language, might be responsible for their giftedness.

## Giftedness

The most basic descriptive model of giftedness is the triadic model (Barbe and Renzulli 1975; Mönks 1985), within which general cognitive abilities, creativity and motivation are all important factors. In this theoretical framework, children are regarded as gifted when they distinguish themselves in these three factors. Furthermore, some contextual factors should, at least to some extent, be in favour of a gifted child too. Such factors might be the right school, a good family environment and supportive age mates. A gifted child is thus one that not only has the right traits, but also the right environment in which these traits can flourish. If the cognitive capacities of a child do not get the opportunity to develop, the child will not be identified as being gifted.

This model formed the basis of different models of giftedness that have been formulated (Gagné 1999; Sternberg 2005), some of which make these personal traits more specific (Heller 2004), whereas others emphasize the negative impact that giftedness might have on the social development of the child. Gifted children do not follow the same development as their peers, which can lead to problems in establishing social contacts with their peers (Kieboom 2007). However, quite independently of the exact theoretical framework adopted, what unites all models is that a gifted child must possess certain cognitive abilities to be regarded as gifted. The exact nature of these cognitive abilities remains unclear, but a general picture can be drawn. Gifted people all seem very well able to recognize patterns and generate creative solutions in at least one domain, such as mathematics, language or science (Sternberg and Davidson 1985).

Few studies have been conducted exploring the common cognitive factor underlying the intellectual development of gifted children. Steiner and Carr (2003) give an overview of the studies that investigated these issues and noticed that ‘despite similar interests […] the fields of gifted education and cognitive development have had little communication’ (p. 216). Still, as Steiner and Carr point out, some topics have been investigated in this specific population; namely their speed of processing, metacognition and problem solving abilities. Gifted children are faster decision makers and they need less time to process incoming information. They often make plans deliberately in order to solve problems, which indicates more metacognitive awareness. Finally, gifted children also tend to choose the right strategy, when confronted with problems that have multiple potential solutions. In order to excel in these domains, Steiner and Carr argue that gifted children require a certain ability to quickly recognize the abstract patterns, which might be necessary to solve problems of different kinds quickly (Hofstadter and Sander 2013; Hong 2013).

## Procedural Memory

Recent psychological literature suggests procedural memory is the part of our cognitive system that is affiliated with the implicit learning and automatization of, amongst other things, abstract rules and motor skills. Procedural memory is responsible for pattern recognition in the auditory as well as the visuo-motoric domain (Packard 2009). Procedural memory differs from declarative memory, as the latter is our storage of more arbitrary information, concrete facts and events.

This declarative/procedural memory distinction plays an important role within language as well. Ullman (2001, 2016) suggests declarative memory can also be regarded as the lexical memory which stores the arbitrary relationships between sounds and their meanings. Procedural memory is domain-general and stores all kind of rules that may work on the elements that are stored in declarative memory, or the lexicon, and the categories that these elements might belong to (Pinker and Ullman 2002). Studies have shown that domain-general procedural memory and linguistic processing are tightly related. Misyak and Christiansen (2012) and Misyak et al. (2010) are two examples of studies that compared individual differences in procedural memory to natural language processing in typically developing populations. Both studies found a correlation between procedural memory capacities and comprehension of relative clauses.

Furthermore, supporting evidence for the role of procedural memory in natural language comes from the specific language impaired (SLI) population (Evans et al. 2009; Hsu et al. 2014; Ullman and Pierpont 2005). SLI is defined as an impairment that is specific to language without having any hearing impairments or general cognitive deficits (Bishop 1997, 2014). Children with SLI generally perform more poorly than an age matched control group when exposed to a task that is typically used to measure procedural learning: the Serial Reaction Time (SRT) task (Lum et al. 2012). In an SRT task, participants see a visual stimulus that repeatedly appears in one of four boxes on a screen. These stimuli appear in a certain pattern of which participants are unaware. Four buttons accompany these boxes and participants press the button that matches the box with the visual stimulus. Typically, participants tend to become faster on trials that behave according to the pattern, whereas they slow down on trials that lack this pattern. The SRT thus proves to be a good measure of procedural memory (Lum et al. 2014).

## The Current Study

As mentioned previously, virtually no studies explored a common cognitive factor underlying the intellectual development of gifted children. Steiner and Carr (2003) propose that gifted children are good at recognizing abstract patterns. Procedural memory underlies pattern recognition and pattern learning. In this study we follow up this link and investigate the hypothesis that gifted children have a better developed procedural memory capacity than their non-gifted peers. We thus expected gifted children to outperform non-gifted children on the SRT task that measures procedural memory.

In addition, as procedural memory and grammatical knowledge are tightly connected with one another, we hypothesize that the higher syntactic knowledge of gifted children is better developed as well because of their enhanced procedural memory. We thus expected gifted children to outperform non-gifted children on a task measuring complex grammatical structures, namely object relative clause comprehension. From an overview by Duinmeijer (2016), it becomes clear that the acquisition of relative clauses starts early, but keeps developing gradually over time in many languages (e.g. Sheldon 1974 for English; Schuele and Nicholls 2000; Thiel et al. 2014 for German; Crain et al. 1990; Belletti and Guasti 2015 for Italian; Friedmann and Novogrodsky 2004, for Hebrew; van Dijk 2014 for Dutch), with children having more trouble with object relative clauses than subject relative clauses (e.g. Kidd et al. 2007; Friedman et al. 2009; Thiel et al. 2014). Several studies, of which some with atypical populations, suggest the trouble with this particular sentence type could arise due to many factors, such as a non-canonical constituent order (MacDonald and Christiansen 2002), a bigger distance between the antecedent and the original position in the sentence structure (O’Grady et al. 2003 for L2 learners) or a larger burden on working memory (Avrutin 2000 for aphasia, Deevy and Leonard 2004 for SLI; Lewis et al. 2006; Seidel 2013, for SLI). Yet, a conclusive explanation for the difficulties with these sentences has not been found, and procedural memory might also be related to a better comprehension of relative clauses (Misyak and Christiansen 2012; Misyak et al. 2010). We thus want to see whether gifted children might show better understanding of object relative clauses, due to a better developed procedural memory. In addition, we expected a correlation between the results on the grammatical task and the SRT task in general.

## Methods

### Participants

To investigate these hypotheses, 25 Dutch speaking gifted children (9 males, 16 females, *M *= 10;6, *SD *= 1;2, *range *=8;6–12;5) were recruited in primary schools in the central southern area of the Netherlands. Children were in grade 3 to grade 6, as this is when most schools start to identify gifted children in their classes. All children participated in a so-called *Plusklas* (‘Plus class’) or *Wetenschapsklas* (‘Science class’) in their schools. Children participate in these classes when regular education is not challenging enough. In the class, children carry out more in-depth exercises once or twice a week. Teachers of the regular class decide who qualify to participate in this educational program. Teachers take both test results of these children in regular education and their own insight and personal experiences with the children into consideration when making this decision. There is no standardized measure that helps identifying children who could participate in these classes. A comparison group consisted of 25 Dutch speaking typically developing children (10 males, 15 females, *M *= 10;9, *SD *=1;2, *range *=8;11–13;3). These children were recruited at primary schools in the same area. These schools offered education for gifted children, but the children in the comparison group did not qualify to participate in this education. Children did not differ significantly in age (*t*(48) = − .66, *p *=0.51) and were matched on grade. Children were assessed with an SRT task to measure procedural memory in the visual-motor domain. A relative clause comprehension (RCC) task measured grammatical knowledge.

### Serial Reaction Time

The SRT task was developed to measure implicit visuo-spatial sequence learning in procedural memory (Lum et al. 2012). Participants were presented a tablet with four boxes on the screen and received a game controller with four buttons that matched these boxes. A smiley appeared in each of the boxes and participants had to press the button that matches the box in which the smiley appears. Unknown to the participants, these smileys appeared in a pattern that started again after every tenth smiley. Reaction times (RTs) of pressing these buttons were measured. Normally, participants tend to press the buttons faster, when they (unconsciously) recognize the pattern underlying the appearance of the smileys. Furthermore, when a test block does not follow the pattern, RTs consequently slow down.

This task consisted of a practice session, in which children could practice with pressing the right buttons on the controller. This session contained 10 random appearances of the smiley. If the children pressed the wrong button 5 or more times, the session had to be completed once more. After practice, four similar patterned blocks of 60 smileys were presented, with every patterned sequence consisting of 10 smileys. This means the same pattern of 10 smileys occurred 6 times each block. During these blocks, participants were expected to become faster at pressing the button, because they came accustomed to the pattern and the task in general. These four blocks were followed by a random block consisting of 60 smileys, in which it is expected that participants become slower. This random block was inserted because participants might become faster at the task only because they get used to it and not because they recognized the specific pattern. If participants indeed recognize a pattern and this causes them to become faster at the task, they should become slower at a random block, as it does not follow the pattern. If participants are not slower at this random block, they only get faster because they accustom themselves to pressing the buttons as fast as possible, but not because they recognize an underlying pattern. After the random block, a final pattern-like block was presented to participants. The pattern in this block was similar to the pattern in the first four blocks.

The task was presented on a tablet (Microsoft Surface 3), with a wired controller (Trust wired gamepad GXT540) attached to it. The explanation was pre-recorded. Results were registered in Eprime (Psychology Software Tools, Pittsburgh, PA). Afterwards, an exit interview was held, within which the experimenter asked whether the participants knew a pattern was underlying the appearance of the smileys. After they were informed there was a pattern, they were asked if they could reproduce it by pressing the buttons of the controller according to what they thought the pattern was.

### Relative Clause Comprehension

An RCC task, adapted from (Duinmeijer 2016), was conducted to measure syntactic knowledge of the children. In this test, participants were presented with a sentence and two pictures. The sentence described only one of these two pictures and participants had to decide which picture matched the heard sentence. Choosing the right picture could only be achieved when a participant understood the sentence correctly. Sentences could be as in (2):

(2a) Dit is de piraat die de clowns slaat.

‘This is the pirate who hits the clowns.’

(2b) Dit is de piraat die de clowns slaan.

‘This is the pirate who the clowns hit.’

**Fig. 1:**
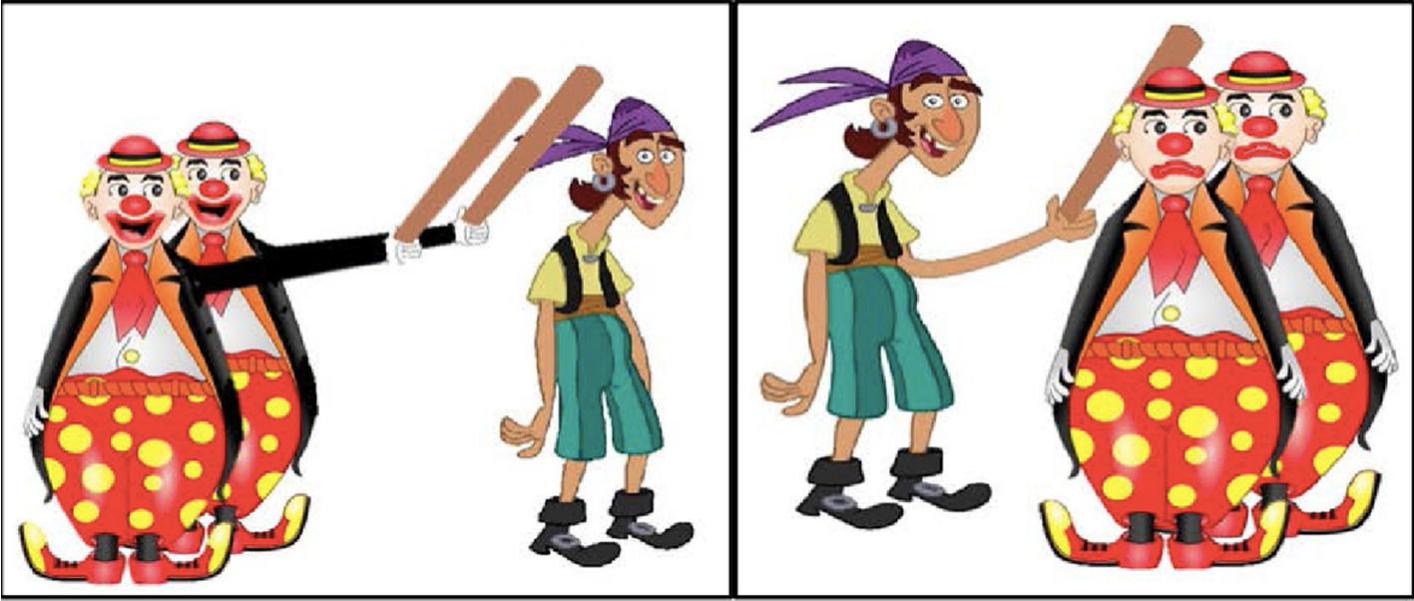
The two pictures between which a participant had to choose, when s/he heard the sentence *Dit is de piraat die de clowns slaan* ‘This is the pirate who the clowns hit’. The left picture was the target

These sentences always started with “*Dit is de … die…”* (‘This is the … who…’), where a person was introduced, for example *piraat* ‘pirate’. The relative clause was either a subject relative clause, where the relative pronoun was the subject in the relative clause (2a), or an object relative clause, where the relative pronoun was the object in the relative clause (2b). The verb in the relative clause was always a transitive verb and could have both an animate subject as well as an animate object, like *hit* ‘slaan’. The interpretation of these sentences crucially depended on the agreement of the verb with a subject, as word order of the two sentences was identical. In (2a), the verb is singular and thus has to agree with the relative pronoun resulting in a subject relative clause. In (2b), the verb is plural and has to agree with the plural *clowns.* The relative clause consequently is an object relative clause. Participants heard only one of these two sentences, but were presented with two pictures that each showed one of these two meanings, as can be seen in Fig. 1.

Determining which picture should be chosen in this test depends on the understanding that interpretation of the sentences is dependent on subject verb agreement. Dutch relative clauses follow an SOV pattern with the critical finite verb appearing in the final position. As this type of sentence typically does not occur that frequently in spoken language, when both arguments of the verb in the relative clause are animate lexical items (Reali and Christiansen 2007), it is not so straightforward to come to the right interpretation of such sentences. Subject verb agreement normally is not necessary for the interpretation of such sentences, because speakers can rely on semantic cues, as the animate constituent is most often also the subject of the sentence. However, to correctly interpret the test sentences, participants had to rely on their syntactic knowledge.

The test consisted of twelve sentences with a subject relative clause (2a) and twelve sentences with an object relative clause (2b). Additionally, six fillers had a relative clause in which both arguments of the verb were singular. These sentences were ambiguous as two arguments could possibly agree with the main verb in the relative clause (3a). When a speaker interprets these sentences as if the relative pronoun agrees with the main verb, we call this a subject interpretation. When a speaker interprets these sentences as if the noun agrees with the main verb, we call this an object interpretation. Furthermore, six fillers contained a relative clause with a passive (3b). Fillers were included to increase the variety in the test.

(3a) Dit is de piraat die de clown slaat.

‘This is the pirate who hits the clown.’/‘This is the pirate who the clown hits.’

(3b) Dit is de piraat die door de clown wordt geslagen.

‘This is the pirate who is hit by the clown.’

All sentences were pre-recorded and played on a laptop in the same order for every participant. Pictures were shown on the screen of the laptop. Participants could either listen to the sentence via headphones or not, depending on their preference. Sentences could be repeated and the participant’s responses were reported on a paper sheet.

### Procedure

The test battery was administered individually to participants during one session at their schools in a quiet room by one experimenter. The tasks were presented in two different orders. Participants either started out with the SRT task, followed by the RCC task or the other way around. A third task, which will not be discussed here, was also administered during the same session. Ethical approval for this study was obtained from the University of Amsterdam and informed passive consent was gained from parents or legal guardians of the children.

### Analyses

All analyses were carried out in R (R Core Team 2015) using the lme4 package (Bates et al. 2015) when necessary. Linear mixed effects models were executed to investigate an effect of learning and group differences in the SRT task. A generalized logistic mixed effect model investigated group differences in the RCC task. In line with (Baguley 2009), we report simple effect sizes and confidence intervals instead of standardized effect sizes and confidence intervals for our mixed effect models. A Pearson’s correlation test was used to investigate a possible relationship between the two tests.

## Results

### Serial Reaction Time

As a first step, the raw data was inspected on outliers. Participants were excluded if they were outliers based on their amount of incorrect presses (> 3 *SD* from the group mean). No participants had to be excluded on these grounds. Furthermore, all incorrect responses were excluded from the analysis, as well as responses that followed an incorrect response, because RTs for these responses could be negatively influenced by a spill over from the previous incorrect response. Additionally, every response by an individual participant that was an outlier (> 3*SD*) when compared to the mean RT of that same participant was excluded too. A total of 6.96% of all trials was removed from the data this way.

**Fig. 2:**
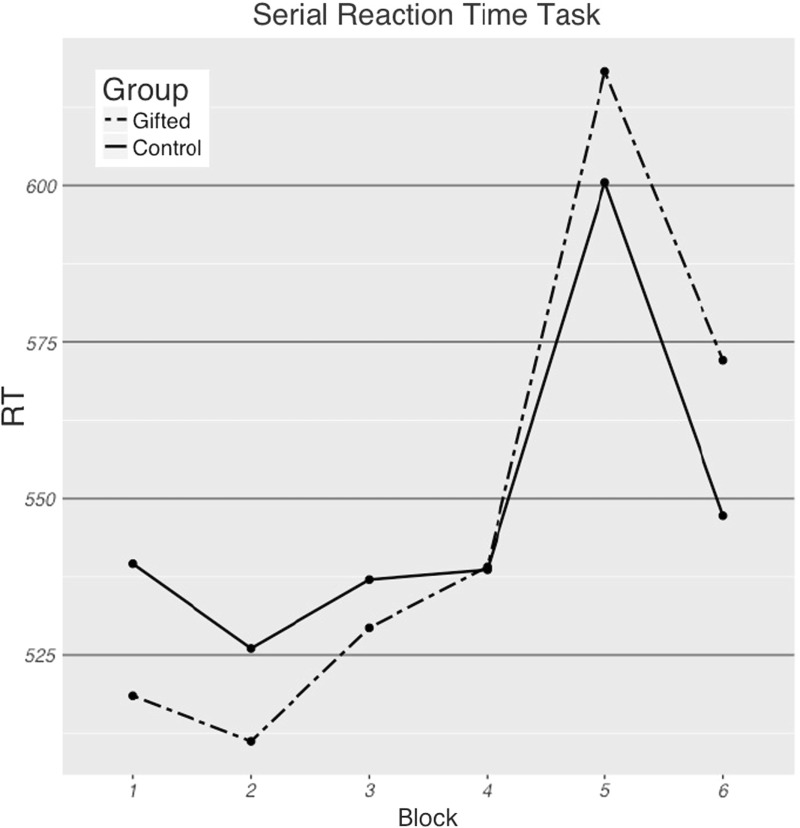
A graph depicting the mean RTs for the two groups. RTs are in ms. Block 5 is the random block

As the test battery was administered to participants in two orders, a first analysis investigated whether this order of tasks during a test session influenced RTs of the last three blocks. We carried out a linear mixed-effects model with mixed effects and orthogonal sum-to-zero coding. This model took reaction time as the dependent variable, order of tasks as a between-participants fixed effect, block as a within-participant fixed effect and participant as a between-participants random effect. Although it was a within-participant fixed effect, block was not included as a random slope for participant, because the model failed to converge when it was included. This analysis did not indicate a significant main effect of the order of the tasks during a session on RTs (point estimate =− 55.52 ms.; 95% confidence interval = − 124.51…13.48 ms.; *t *=− 1.577), nor a significant interaction between this order of testing and block number on RTs (point estimate =− 1.863 ms.; 95% confidence interval = − 19.06 …15.33 ms.; *t *=− 0.21). This does not indicate that order influenced the possible differences in RTs between the different blocks of the test. Results from participants who started with the SRT and the results from those who finished with the SRT were thus analysed together. These combined results for the two groups can be seen in Fig. 2.

Further analyses investigated whether there was a main effect of block on RTs in the last 3 blocks, whether there was an effect of group on these same RTs and if there was an interaction between the two. A linear mixed-effects model with orthogonal sum-to-zero coding was carried out. This model took reaction time as the dependent variable, group as a between-participants fixed effect, block as a within-participant fixed effect and participant as between-participants random effect. Although it was a within-participant fixed effect, block was not included as a random slope for participant, because the model failed to converge when it was included. This model indicated a significant main effect of block type on RTs, as participants were slower during the random block than the average of the two patterned blocks (point estimate = 53.47 ms., 95% confidence interval = 44.88…62.06 ms., *t *=12.20), but no main effect for group (point estimate = − 14.45 ms., 95% confidence interval = − 84.97…56.06 ms., *t *=− 0.402) and no interaction between group type and block (point estimate = − 1.888 ms., 95% confidence interval = − 15.29…19.06 ms., *t *=0.215).

### Relative Clause Comprehension

**Fig. 3:**
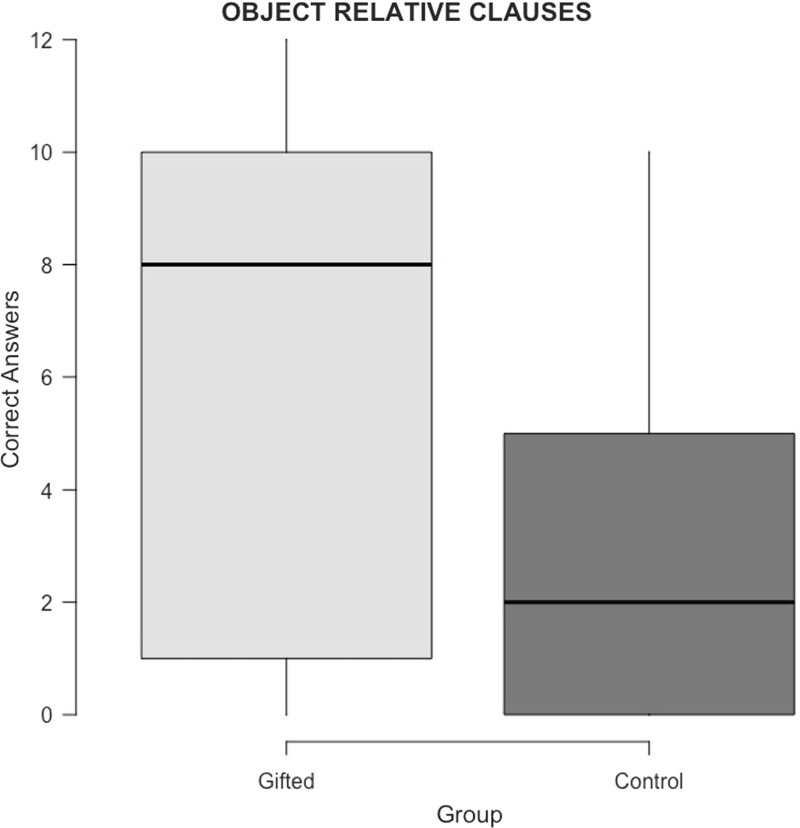
A box-plot depicting the amount of correct answers for the items of the RCC task that tested comprehension of object relative clauses

The RCC task consisted of 36 items of which 12 items contained an object relative clause and 12 items contained a subject relative clause, which were the types of sentence that were of interest. Additionally, 6 fillers contained a passive relative clause and 6 more fillers a relative clause that contained two singular arguments. Box-plots in Fig. 3 show the results from the items with an object relative clause. Table 1 shows the results and scores for all sentence types. For ambiguous relative clauses, scores reflect the number of times a participant choose a subject interpretation. Participants were excluded if they gave a correct answer in half or less than half of the fillers with a passive relative clause. No participants had to be excluded on these grounds.

**Table 1:** Scores from the RCC task and comparisons

Task	Gifted			Control		
	M (%)	SD	Range	M (%)	SD	Range
Object relative clauses	6.08 (51%)	4.42	0–12	2.92 (24%)	3.01	0–10
Subject relative clauses	11.72 (98%)	0.46	11–12	11.40 (95%)	0.87	9–12
Double singular relative clauses	5.68 (95%)	0.56	4–6	5.60 (93%)	0.58	4–6
Passive relative clauses	5.76 (96%)	0.44	5–6	5.44 (91%)	0.47	4–6

The first two conditions contained 12 items, the latter contained 6 items

As the results for all types of filler sentences were at ceiling for both groups, only an analysis was run for the items containing an object relative clause. A generalized logistic model with mixed effects and orthogonal sum-to-zero coding was conducted. This model took the clause comprehension score as dependent variable, group as a between-participants fixed effect, participant as a between-participants random effect and item as a within-participants random effect. The model showed a significant main-effect of group (*OR *= 7.12, 95% confidence interval = 1.77… 31.92, *z* = 2.759, *p* = 0.006).

### Correlations

Finally, scores from the different tasks were correlated. For the SRT task, a difference score per participant was computed between block 4 and block 5 by subtracting the mean RT of block 4 from the mean RT of block 5 for each participant. The difference between these blocks was chosen, because it is most indicative of a learning effect during test phase The relapse in RTs when the random block was presented shows whether participants were habituated to the pattern. A bigger difference score indicates a bigger learning effect. For the RCC task, the amount of correct answers on the object relative clause was taken. Results from a Pearson’s correlation analysis did not show a significant correlation between the SRT task and the RCC task for both groups combined (*r *= − 0.219, *p *=0.127). As we expected a correlation between procedural memory and relative clause comprehension in general and not for one specific group, we did not carry out any further analyses on separate groups.

## Discussion

This study examined whether gifted children and their non-gifted age peers differed with regard to their procedural memory capacities. We predicted that the former would outperform the latter in different domains that are related to this type of memory. In order to investigate these questions, an SRT task was used to measure procedural memory and an RCC task measured syntactic comprehension.

The results from the different tests are noteworthy in several ways. First of all, the groups did not perform differently on the SRT task, in which they both showed significantly slower RTs during the random block, which reflects implicit learning of the sequence. Results from this SRT task thus do not provide evidence that gifted children possess a better developed procedural memory in the visuo-motoric domain. A difference between the two groups was observed in the RCC task. The gifted children showed a significantly better understanding of object relative clauses compared to the comparison group. These findings thus indicate the gifted children have a better syntactic processing capacity. However, this probably is not caused by a general procedural memory advantage, because there was no significant correlation between the RCC task and the SRT task.

One alternative explanation for the advantage of the gifted children on the syntactic comprehension task could be that these children might receive different linguistic input and, as a result, are better at processing this type of sentences. Hulstijn (2017), for example, shows higher educated people use more object relative clauses in Dutch than lower educated people do. Gifted children are possibly exposed more to the language of higher educated people and thus to this type of sentences, for example, because they read more often. As seen in the different models of giftedness (Barbe and Renzulli 1975; Mönks 1985; Heller 2004; Gagné 1999), these children not only have certain capacities, but are also in the right environment in order to become ‘gifted’. Although we did not collect any of such background data, these results might indicate that such an environment plays an important role; gifted children do not distinguish themselves on the basis of their procedural memory, but have more experience with the language of higher educated people.

Another alternative explanation might be that gifted children make better use of their meta-cognitive skills in order to comprehend object relative clauses. Steiner and Carr (2003) argue gifted children employ such skills to complete tasks or solve problems more efficiently than non-gifted children. Perhaps meta-cognitive skills that are specific to language provide gifted children a better opportunity to process and acquire these sentences, as such explicit meta-linguistic knowledge could play an important role in the language acquisition process in general (Ellis 2015).

In sum, this study fails to show gifted children differ in their procedural memory capacities, but it does show they differ in their syntactic comprehension. This might happen, because they have been exposed more to the type of language containing object relative clauses and are more familiar with them, whereas children that do not grow up in such environments are less exposed to this type of sentences. Gifted children could also possess more meta-linguistic knowledge and make better use of this knowledge when acquiring and using object relative clauses. Further research should show whether some other general cognitive mechanism, meta-linguistic knowledge, different linguistic input or perhaps another factor is responsible for the difference in syntactic comprehension between gifted and non-gifted children.
